# Management of Acetabular Bone Loss in Hip Revision Arthroplasty: Case Series Presentation

**DOI:** 10.7759/cureus.45432

**Published:** 2023-09-18

**Authors:** Adrian Cursaru, Sergiu Iordache, Mihai Costache, Bogdan Serban, Mihnea Popa, Bogdan Cretu, Catalin Cirstoiu

**Affiliations:** 1 Orthopedics and Traumatology, University Emergency Hospital, Bucharest, ROU; 2 Orthopedics and Trauma, Carol Davila University of Medicine and Pharmacy, Bucharest, ROU; 3 Orthopedics and Traumatology Department, University Emergency Hospital, Bucharest, ROU; 4 Orthopedics, Bucharest Emergency University Hospital, Bucharest, ROU

**Keywords:** acetabular bone loss, acetabular reconstruction, acetabular cage, revision surgery of the hip joint, total hip arthroplasty: tha

## Abstract

Considering the increase in life expectancy in the general population and the need for a more active lifestyle, total hip arthroplasty has become an absolutely necessary surgical intervention to maintain these desired results.

Along with the evolution of medicine and the increase in the quality and performance of the materials used to make prostheses, the number of patients who benefit from total hip replacement is constantly increasing, and proportionally, the number of patients who will require revision arthroplasty is increasing.

Before discussing the need for hip arthroplasty revision, it is necessary to carry out a rigorous clinical and imaging examination for differential diagnosis with other pathologies such as low back pain, the presence of bone or soft tissue tumors, arterial occlusions and claudication, or other systemic diseases.

One of the biggest challenges for the orthopedic surgeon in planning a hip revision is the compensation of the remaining acetabular bone defect, either as a result of the osteolysis process or following the process of removing the acetabular component, which in some cases can lead to severe bone loss that is difficult to anticipate in the preoperative planning.

In this paper, we will present the short-term results of the use of reinforcement cages fixed with screws and cemented retentive acetabular cups in the case of hip revisions with extensive bone loss.

The discussions that derive from the presented series of cases are related to the use of reinforcement cages, which are based on the principle of primary stability obtained with the help of screw fixation but whose risk of osteolysis and implant fixation damage is greater than in the case of implants that also associate biological integration at bone level.

The use of reinforcement cages together with the retentive acetabular cup in the case of elderly patients with associated comorbidities, a moderate level of physical activity, and severe muscle insufficiency at the hip level as a result of not using the affected pelvic limb is still a viable solution that allows the patient to walk immediately after the surgery, avoiding the risk of dislocation (especially in patients who use the posterolateral approach) and avoiding morbidity induced by prolonged bed rest.

## Introduction

Considering the increase in life expectancy in the general population and the need for a more active lifestyle, total hip arthroplasty has become an absolutely necessary surgical intervention to maintain these desired results. Total hip arthroplasty in the cemented or uncemented version continues to offer a high degree of patient satisfaction with an excellent functional result in terms of pain and joint mobility. The excellent functional results of total hip arthroplasty, with a success rate of over 95% after 10 years of follow-up, have included this surgical intervention in the top surgical procedures with the highest degree of satisfaction from the patient [1,2].

Along with the evolution of medicine and the increase in the quality and performance of the materials used to make prostheses, the number of patients who benefit from total hip replacement is constantly increasing. Total hip arthroplasty has seen a constant improvement regarding the design of the implants, the type of fixation, and the quality of the materials, leading to a decrease in the revision rate [3]. The use of hip arthroplasty in young patients, the sustained activity, and the increase in life expectancy mean that approximately 10%-15% of total hip arthroplasty patients require component revision after 20 years. Osteolysis and aseptic loosening, resulting from failure of bearing surfaces, are considered to be the most common reasons for revision hip arthroplasty [4]. Other causes are represented by wear, implant failure or breakage, or causes that lead to an arthroplasty revision in a much shorter time, such as periprosthetic infection, recurrent dislocations, periprosthetic fractures, or mechanical loosening [5-7].

Another important component that can lead to the early loosening of the implant or recurrent dislocations is related to the surgical technique and the correct positioning of the implant [8].

The use of non-steroidal anti-inflammatory drugs, smoking, and increased body mass index are cited as potential risk factors in the early deterioration of total hip arthroplasty [9,10].

Indication for THA revision

Pain is the main symptom that leads to the presentation of the patient with total hip arthroplasty to the orthopedic surgeon. Before discussing the need for hip arthroplasty revision, it is necessary to carry out a rigorous clinical and imaging examination for differential diagnosis with other pathologies such as low back pain, the presence of bone or soft tissue tumors, arterial occlusions and claudication, or other systemic diseases.

The exclusion of another cause of pain does not automatically lead to establishing the indication for revision and must take into account the degree of pain and whether it is improved by the use of NSAIDs, the degree of physical activity of the patient, the associated pathologies, and the patient's expectations, bearing in mind that revisions are laborious surgical interventions with increased operating time, significant blood loss, and numerous peri- and postoperative complications compared to primary hip arthroplasty.

Jie J. Yao et al., in a retrospective study that included 4532 patients who benefited from revision hip arthroplasty, observed an increase in long-term mortality. Mortality compared to the general population was increased in the case of patients who required revision for periprosthetic infection or periprosthetic fracture, while in the case of patients with aseptic loosening, the mortality was similar to that of the general population [11].

After excluding a secondary cause or a septic process, pain in the case of patients with aseptic loosening of the acetabular, femoral component, or both documented by imaging is the main indication for revision.

Limitation of joint mobility unaccompanied by pain is not an indication for component revision and may benefit from non-surgical therapeutic measures.

A loose acetabular component usually produces pain in the groin, whereas a loose femoral component may cause pain in the thigh or knee.

Acetabular bone loss: a challenge for the orthopedic surgeon

One of the biggest challenges for the orthopedic surgeon in planning a hip revision is the compensation of the remaining acetabular bone defect, either as a result of the osteolysis process or following the process of removing the acetabular component, which in some cases can lead to severe bone loss that is difficult to anticipate in the preoperative planning.

The preparation of a hip revision must include a careful screening of associated infections, a thorough clinical examination with observation of the gait and recording of a pathological gait (abductor muscle weakness), correct imaging documentation using radiographs of the pelvis in the antero-posterior view, Judet views for a better assessment of the anterior and posterior column of the pelvis, a CT scan, possibly with 3D reconstruction, for a more accurate assessment of the available bone capital, and, as far as possible, the assessment of the remaining bone capital after implant removal [12]. Novel technologies, including dynamic CT-based micromotion analysis and advances in CT and RSA technologies, may prove helpful in determining bone ingrowth and the degree of component migration.

Equally, the orthopedic surgeon must take into account the patient's age, sex, bone mineral density and factors that can lead to a decrease in bone mineral density, such as the use of systemic glucocorticoids. In the case of patients with significant bone loss at the acetabular level, it is mandatory to perform a rigorous neurological and vascular examination, considering the fact that the risk of iatrogenic nerve damage in the case of acetabular reconstructions is high.

All the previously mentioned factors must lead to a good preparation of materials and implants, such as the expected cup size, bearing couple, implant constraint, and additional materials needed (mesh, acetabular augments, cages, bone grafts, or custom-made acetabular components).

Preparation of larger acetabular cups, 70-75 mm, may be necessary for extensive bone defects.

For the evaluation of acetabular bone defects, the Paprosky classification is used, described in 1994, and this classification system was useful in predicting the integrity of major anatomical landmarks, including the teardrop, ischial bone loss, the ilioischial line (Kohler's line), and the degree of superior migration of the cup [13].

Implant selection

Choosing the right implant for the patient who requires hip arthroplasty revision is a challenge for the orthopedic surgeon, who must consider the correct preoperative clinical and imaging evaluation so as to provide an adequate primary fixation and allow full weightbearing immediately post-operatively with durability and long-term functionality [14].

From a biomechanical point of view, acetabular reconstruction must pursue two main objectives. The first objective, especially for cages and ring reconstruction, is to obtain a rigid, stable fixation that ensures the resistance of the implant over time, taking into account the absence of a secondary, biological fixation. These mechanical constructs should be placed in an optimal biomechanical position (inferior and medial instead of superior lateral) to optimize long-term implant durability.

The second objective considers the restoration of the native acetabular center of rotation, leading to improved hip stability and abductor function, as well as optimized hip range of motion and biomechanics [15].

Hemispherical acetabular shells

A wide range of hip revisions with acetabular bone defects can be treated using hemispherical acetabular shells. The golden rule when using this type of implant is to ensure a bone contact of at least 50% [16-18].

However, a large hemispherical socket has limitations and is not adequate for all types of bone loss, especially in the case of patients with a small antero-posterior acetabular diameter. The transformation of this elliptical defect into one that allows obtaining a press fit fixation is done in many cases with the sacrifice of the anterior and posterior columns [19].

Allograft bone augmentation and augments

Allograft bone augmentation combined with various acetabular cups can be used for acetabular bone loss. The reconstruction principles for impaction grafting are a combination of both mechanical and biological. However, the use of cancellous autografts presents an increased septic risk, which will have to be removed with the treatment of PJI.

An important step in filling acetabular defects is represented by the use of augments that can provide the excellent anterosuperior to posteroinferior “pinch” fixation, that is crucial for stable socket fixation. Their disadvantage is related to the increased cost compared to the use of autografts and the fact that they do not form bone capital, which complicates a potential revision [20-22].

Another option for a larger medial wall defect is a smaller acetabular cup as medial support, or a cup-on-cup configuration. The medial cup is impacted first to achieve medial support. The second cup is then reamed and impacted with additional screw fixation through both cups.

Cages and rings

Acetabular cages are useful in the case of patients with loss of more than half of the acetabular surface, bone loss at the level of the anterior and posterior columns, or pelvic discontinuity. Reinforcement cages ensure stability for the acetabular construction and transmit the load to the host bone through the cage for bone grafts, cups, and augments.

Two main kinds of acetabular cages are described in the literature: acetabular roof rings and antiprotrusio cages. Acetabular roof rings may or may not have a hook for cotyloid notches and usually do not have flanges for the ilium, whereas antiprotrusio cages are characterized by double flanges for the ilium and the ischium.

Cup-cage constructs

They are useful in the case of patients with massive acetabular bone defects and even pelvic discontinuity. The surgical technique is based on the initial fixation of the cup in the remaining bone defect so as to allow a future secondary fixation with bone ingrowth, and then the cage is fixed over the cup so as to provide increased primary stability, thus forming a stable skeleton for the acetabular cup.

The use of cup-cages is difficult, especially in the case of patients with limited bone reserve, but it is especially useful in the case of patients with pelvic discontinuity who cannot afford to wait for a custom-made implant [23].

Patient-specific implants

Patient-specific devices require extensive planning with engineers before surgery to create an implant that will span and reinforce the pelvic deficiency. As we mentioned before, the use of cup-cage or triflange implants is a viable alternative for patients with significant acetabular defects or pelvic discontinuities. The indication for choosing patient-specific implants remains for patients with a small pelvis, in which case an adequate fixation cannot be obtained using the previously mentioned implants [24-29].

The disadvantages of this type of implant are related to the cost of manufacture, the time required for its manufacture, and the tendency to lateralize the center of rotation.

In the following, we will present a series of four hip revision cases from the Department of Orthopedics and Traumatology at the University Emergency Hospital of Bucharest.

## Case presentation

Case 1

**Figure 1:**
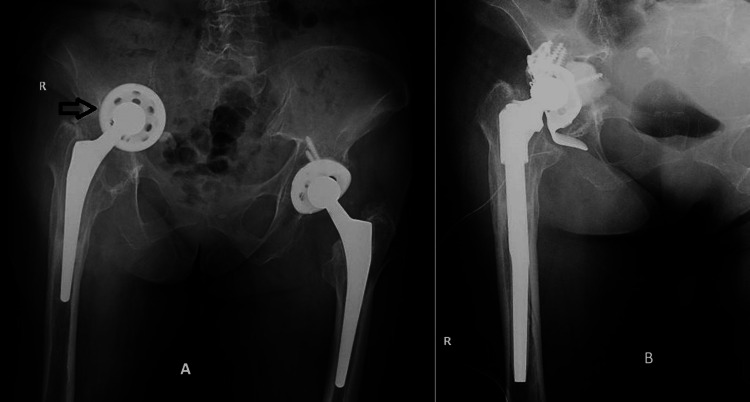
A. Preoperative antero-posterior pelvic X-ray. B. Postoperative right hip X-ray.

A 74-year-old female patient presented to the orthopedic department of the University Emergency Hospital of Bucharest with pain and total loss of mobility at the level of the right hip. Pain started approximately 12 months ago, with no recent traumatic history. The patient had a shortening of approximately six centimeters at the level of the right pelvic limb with a marked limitation of joint mobility. From the personal pathological history, we recall that the patient benefited from right hip arthroplasty with uncemented total prosthesis in March 2018 and arthroplasty with uncemented total prosthesis in October 2018, hypothyroidism in substitutive treatment, mitral insufficiency grade 2, tricuspid insufficiency grade 2, and mild normochromic normocyttic iron-deprived anemia. At the time of admission, the patient did not present a biological inflammatory syndrome or inflammatory changes at the level of the surgical approach. For this reason, after the preoperative preparation, it is decided to revise the arthroplasty by practicing the extraction of the components through a posterolateral iterative approach. The remaining bone defect at the acetabular level, Paprosky IIIB with supero-medial cup migration (Figure 1A), was compensated using a 56-mm reinforcement cage fixed with screws and a cemented retentive acetabular cup. The bone defect at the femoral level was compensated using an uncemented modular revision prosthesis with a 210 mm/14 mm diaphyseal component and a standard 35 mm metaphysis with a CCD angle of 135 degrees (Figure 1B).

The local and general postoperative evolution was favorable without notable complications with the start of walking 48 hours postoperatively with initial partial support on the operated limb.

Case 2

**Figure 2:**
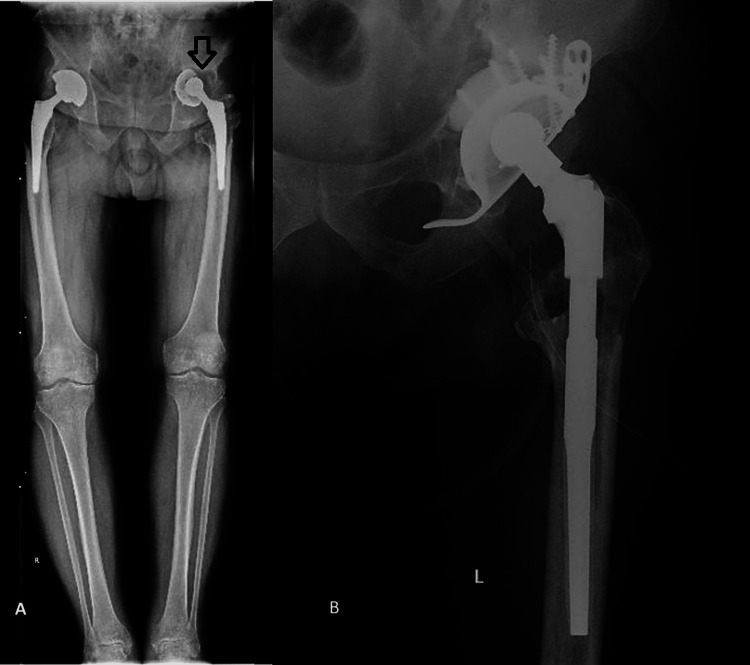
A. Preoperative full-leg X-ray. B. Post-operative left hip X-ray.

A 75-year-old patient known to have had arthroplasty with an uncemented total hip prosthesis at the level of the left hip since June 2012 and arthroplasty with a total uncemented hip prosthesis at the level of the right hip since March 2021. The patient presented himself at the orthopedic clinic of University Emergency Hospital of Bucharest with progressive onset pain and aggravation of symptoms in the last nine months, without a traumatic history. From the personal pathological antecedents, we note essential high blood pressure treatment, vitiligo, and chronic venous insufficiency. Following the preoperative clinical and imaging evaluation, the patient does not present any inflammatory syndrome or other associated pathologies that would contraindicate the surgical intervention. Radiologically, marked areas of osteolysis are described at the acetabular level with migration of the acetabular cup, Paprosky IIIB. Also, at the femoral metaphyseal level, important areas of osteolysis are described (Figure 2A).

The revision surgery was performed with the patient in the lateral decubitus through an iterative postero-lateral surgical approach. After extracting the components, reconstruction was performed at the acetabular level with a reinforcement cage fixed with screws, diameter 70 mm, a cemented retentive acetabular cup, and at the level of the femur, revision was performed using a modular revision prosthesis, with the diaphyseal component 210 mm/16 mm, the uncemented and standard metaphyseal component 35 mm, and a CCD angle of 135 degrees (Figure 2B). The local and general postoperative evolution was favorable; the patient resumed walking with progressive partial support 24 hours postoperatively without notable local or general complications.

Case 3

**Figure 3:**
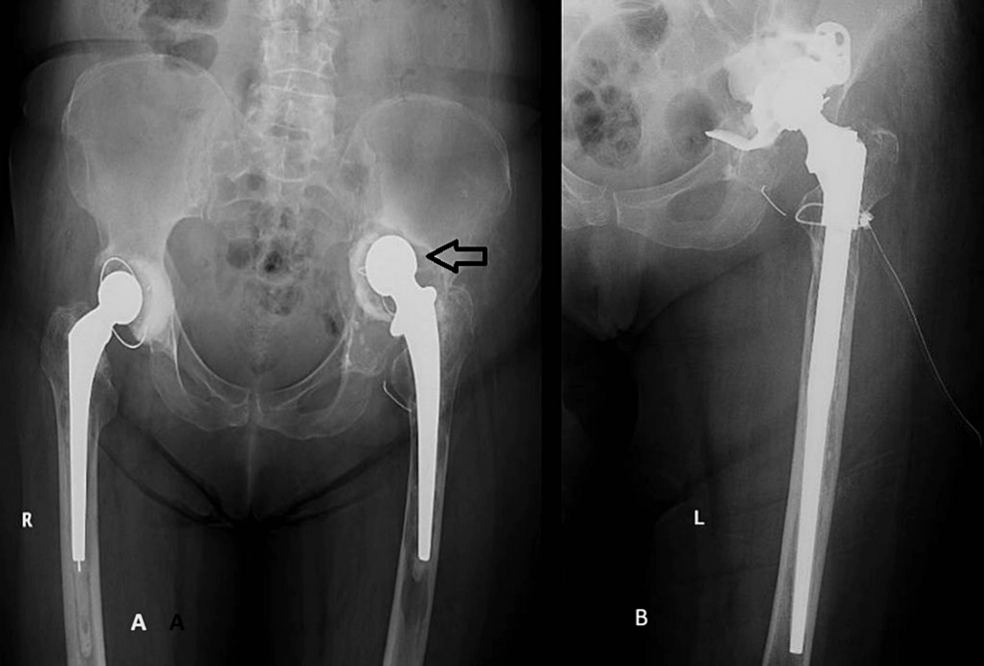
A. Preoperative antero-posterior pelvis X-ray. B. Postoperative left hip X-ray.

A 78-year-old patient known to have had arthroplasty with a cemented total prosthesis on the right hip since April 1996 and a total cemented prosthesis on the left hip since January 2008. The patient presented to the orthopedic clinic of the University Emergency Hospital of Bucharest with pain in active and passive mobilization of the left hip and shortening of the left pelvic limb without a history of trauma. The pain started about a year ago with a progressive worsening of symptoms. From the personal pathological antecedents, we mention hypertension under treatment, mitral insufficiency degree 2, and tricuspid insufficiency degree 2. Biologically, the patient has ESR 18 (N.value: 5-10 mm/h) but without other changes in the inflammatory markers. During the preoperative infectious screening, no signs of infection are detected. Radiologically, the patient shows massive areas of osteolysis at the acetabular level with migration of the acetabular cup superior-medially (Paprosky IIIB) (Figure 3A). Also, at the femoral level are shown areas of osteolysis with signs of decimation of the femoral component.

Surgical intervention is performed, and the ablation of the decimated prosthetic components is performed through a posterolateral iterative approach. At the acetabular level, reconstruction is performed using a 50-mm-diameter reinforced cage and a cemented retentive cup. At the femoral level, considering the important areas of osteolysis, we used a modular revision prosthesis with a diaphyseal component of 250 mm or 14 mm, a standard metaphyseal component of 35 mm, and a CCD angle of 135 degrees (Figure 3B). The post-operative evolution of the patient was favorable, without local complications, with only moderate anemia (Hgb-7.4 mg/dl), for which two units of erythrocyte mass were administered. The patient mobilized 48 hours after surgery with progressive partial support and without other notable complications.

Case 4

**Figure 4:**
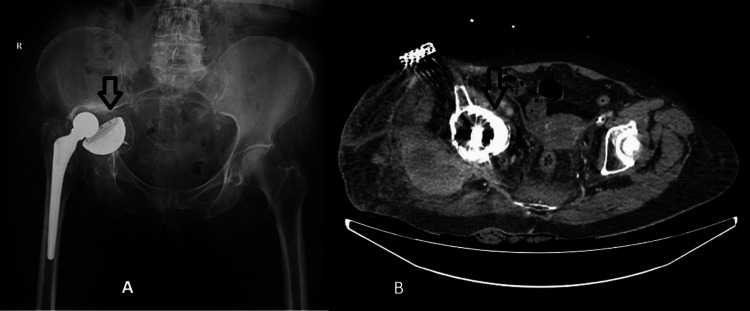
A. Preoperative antero-posterior pelvis X-ray. B. Preoperative pelvis CT-scan-axial view.

A 73-year-old female patient known to have had arthroplasty with an uncemented total prosthesis at the level of the left hip since February 2003 with a history of trauma by falling from approximately 1 m was diagnosed in another hospital with a periprosthetic fracture at the acetabular level and with the endopelvic protrusion of the acetabular cup four months before the presentation in the orthopedic department of the University Emergency Hospital of Bucharest. From the personal pathological antecedents, we mention high blood pressure under treatment, type II insulin-dependent diabetes mellitus, and viral infection with the hepatitis C virus. Radiologically, the endopelvic migration of the acetabular cup with a superior-external orientation and dislocation of the prosthesis can be detected (Figures 4A, 4B). Clinically, the patient presents spontaneous pain during active or passive mobilization maneuvers of the right pelvic limb, with a shortening of the limb of approximately 4 centimeters.

**Figure 5:**
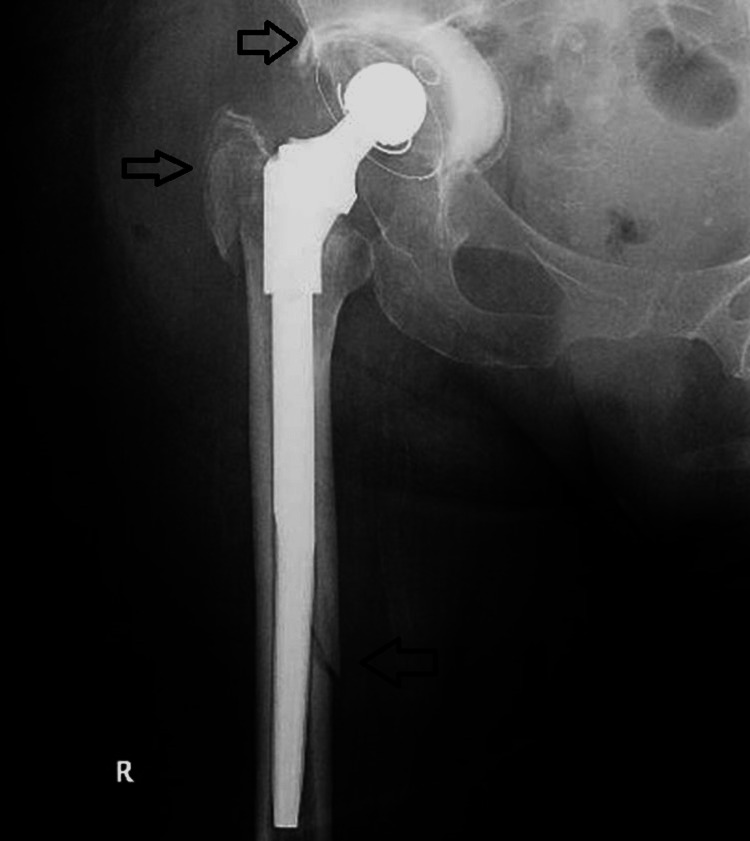
Postoperative right hip X-ray.

After the clinical and imaging evaluation, surgical intervention is performed through a postero-lateral approach, with the patient in the lateral decubitus practicing the extraction of the prosthetic components. The patient becomes hemodynamically unstable during the surgical intervention with a drop in blood pressure, which is why, in order to shorten the operative time and bleeding, it is decided to reconstruct at the acetabular level with the help of a cemented retentive cup with a diameter of 58 mm and at the femoral level with the help of a modular revision prosthesis with a diaphyseal component of 210 mm/14 mm, a metaphyseal component of 35 mm, and a CCD angle of 135 degrees (Figure 5). Intraoperatively, an iatrogenic fracture line is detected at the level of the femoral diaphysis in the middle third and at the level of the greater trochanter. Post-operatively, the patient remains unstable and is transferred to the intensive care unit for advanced life support. After 72 hours, the patient is hemodynamically and respiratory stable, which is why she is transferred to the orthopedic clinic for the continuation of specialized treatment. The post-operative evolution is slowly favorable, with the start of mobilization approximately five days post-operatively without support on the operated pelvic limb for six weeks.

## Discussion

Our clinical algorithm, before performing the hip revision surgery, considered the patient's state of health, his life expectancy, the patient's expectations, his level of activity, and the reason why the revision of the arthroplasty is necessary. Also, we considered the situation when the patient cannot mobilize, and the restoration of the hip joint becomes mandatory to resume walking and the avoidance of morbidity and mortality induced by prolonged bed rest, along with the availability of implants and reconstructive solutions necessary for each individual patient as soon as possible.

The discussions that derive from the presented series of cases are related to the use of reinforcement cages, which are based on the principle of primary stability obtained with the help of screw fixation but whose risk of osteolysis and implant fixation damage is greater than in the case of implants that also associate biological integration at bone level.

Another discussion is related to the use of retentive acetabular cups versus semi-retentive cups. The degree of joint mobility is lower in the case of the retentive cup, with an increase in the risk of decimation due to the greater forces expressed at the cement-implant interface, but with a lower dislocation risk than the semi-retentive cups, especially in the conditions of using a posterolateral approach. 

We remind the patients to come to our hospital after at least three months of bed rest or minimal walking without support on the affected pelvic limb. Of course, the final decision to use reinforcement cages and the retentive cup took into account the age of the patients, approximately 75 years old, who at admission presented moderate to severe muscle atrophy.

Studies on the medium- and long-term durability of the reinforcement cages show moderate results because of the lack of a bone ingrowth surface. Furthermore, this type of implant represents a viable alternative in the treatment of extensive acetabular defects, especially in the case of elderly patients who require a hip revision as quickly as possible, with the resumption of early mobility and full weightbearing, being patients who can no longer wait for a custom-made implant or where the use of bone autografts, together with the increased septic risk, do not allow another surgical intervention to repair a PJI [30].

We mention that in one of the four presented cases, more precisely in the presented case number four, only a retentive acetabular cup of increased dimensions, 58 mm, was used. Although the initial plan was for reconstruction with the help of a reinforcement ring, the degree of hemodynamic instability of the patient during the surgical intervention required a shortening of the operative time and the bleeding. 

According to many authors, the use of retentive acetabular cups in hip revisions in elderly patients with muscle insufficiency and a low degree of physical activity represents a good solution with good results in the short and medium term [31,32].

Although the presented cases do not benefit from a follow-up period longer than one year, according to the supporting evidence, the use of reinforcement cages remains a viable solution for patients with extensive bone loss at the acetabular level. This is in accordance with the results presented by Alexander Ewars et al. in a clinical study that highlighted a 77.7% survival rate after 10 years [33].

## Conclusions

The choice of a reinforcement cage together with a cemented retentive cup is based on, in addition to the immediate availability of the implant compared to other reconstruction solutions, the age of the patients, which is approximately 75 years old, the associated comorbidities, but most importantly, the long evolution of the symptoms, with significant gait deformations, prolonged bed rest, and moderate to severe muscle atrophy in the hip muscles.

The use of reinforcement cages together with the retentive acetabular cup in the case of elderly patients with associated comorbidities, a moderate level of physical activity, and severe muscle insufficiency at the hip level as a result of not using the affected pelvic limb is still a viable solution that allows the patient to walk immediately after the surgery, avoiding the risk of dislocation (especially in patients who use the posterolateral approach) and avoiding morbidity induced by prolonged bed rest.
